# Supplementary material for: Nexus fermions in topological symmorphic crystalline metals
Source: Sci Rep. 2017 May 10;7:1688. doi: 10.1038/s41598-017-01523-8 (PMC5431971; doi:10.1038/s41598-017-01523-8)
Supplement: Supplementary file 1 — Supplementary Information [file 41598_2017_1523_MOESM1_ESM.pdf]

Supplementary Information:

Nexus fermions in topological symmorphic crystalline metals

Guoqing Chang*,[1,2] Su-Yang Xu*†,[3] Shin-Ming Huang,[4] Daniel S. Sanchez,[3]
Chuang-Han Hsu,[1] Guang Bian,[3] Zhi-Ming Yu,[5,6] Ilya Belopolski,[3]
Nasser Alidoust,[3] Hao Zheng,[3] Tay-Rong Chang,[7] Horng-Tay Jeng,[7,8]
Shengyuan A. Yang,[6] Titus Neupert,[9] Hsin Lin†,[1,2] and M. Zahid Hasan†[3]

[1]*Centre for Advanced 2D Materials and Graphene Research Centre National University of Singapore, 6 Science Drive 2, Singapore 117546*

[2]*Department of Physics, National University of Singapore, 2 Science Drive 3, Singapore 117542*

[3]*Laboratory for Topological Quantum Matter and Spectroscopy (B7), Department of Physics, Princeton University, Princeton, New Jersey 08544, USA*

[4]*Department of Physics, National Sun Yat-Sen University, Kaohsiung 804, Taiwan*

[5]*School of Physics, Beijing Institute of Technology, Beijing 100081, China*

[6]*Research Laboratory for Quantum Materials, Singapore University of Technology and Design, Singapore 487372, Singapore*

[7]*Department of Physics, National Tsing Hua University, Hsinchu 30013, Taiwan*

[8]*Institute of Physics, Academia Sinica, Taipei 11529, Taiwan*

[9]*Princeton Center for Theoretical Science, Princeton University, Princeton, New Jersey 08544, USA*

* These authors contributed equally to this work.

† Corresponding authors (emails): suyangxu@princeton.edu, nilnish@gmail.com, mzhasan@princeton.edu

This file includes:

SI A. Fermi surface of the new TM

SI B. Class α triply-degenerate fermions in WC Family

SI C. Materials hosting Class β triply-degenerate fermions

SI D. Effective three-band Hamiltonian in the presence of M_z symmetry

SI E. Effective three-band Hamiltonian in the absence of M_z symmetry

SI F. Solution of Landau Levels

Fig. S1 to S5

SI A. Fermi surface of the new TM

In order to understand the band structure of the new topological semimetal, we study its Fermi surface topology. We study the constant energy contours in the (k_y,k_z) plane at three different energies E_+, E_0, and E_-, that are above, below, and at the energy of the triply-degenerate node, respectively. Consider first a pair of type-I triply-degenerate nodes as shown in Figs. S1**a**,**b**. At E_+, a large electron pocket (α-pocket) enclosing a pair of projected triply-degenerate fermions, and a smaller electron pocket surrounding individual triply-degenerate fermions comprises the Fermi surface. Studying the evolution of both electron pockets as one tunes the binding energy to E_- reveals that both pockets shrink, as expected. However, now we observe that the α-pocket (red) is in between the pair of triply-degenerate fermions while a hole pocket (yellow) emerges and surrounds individual triply-degenerate fermions. At the energy of the triply-degenerate fermions, E_0, only a α-pocket is observed that connects the pair of triply-degenerate fermions. The two observed pockets always have a point of degeneracy, which is due to the two-fold degenerate band along the z-axis. To contrast the Fermi surface behavior of emergent type-I triply-degenerate fermions with those of type-II, we will now study the Fermi surface arising from type-II triply-degenerate fermions, Fig.S1 **c**. One clear distinguishing feature in the series of constant energy contours shown in Fig.S1 **d** (E_+ to E_-) is that there are three Fermi surfaces, which consist of both electron (red) and hole (yellow) pockets at all energies. Furthermore, by scanning through the binding energies, it becomes evident that the electron pocket (α-pocket), composed of two closed contours, encloses the pair projected type-II triply-degenerate fermions at E_+ and then shrinks to occupy the space in between the pair of triply-degenerate fermions at E_-. The outer electron pocket is degenerate with the hole pocket at E_+, which then become disconnected below E_0, where now the hole pockets are enclosing the projected type-II triply-degenerate fermions. At energy and momentum space location of the projected type-II triply-degenerate fermions, the three pockets become degenerate, which is consistent with the three-fold and type-II nature of these fermions.

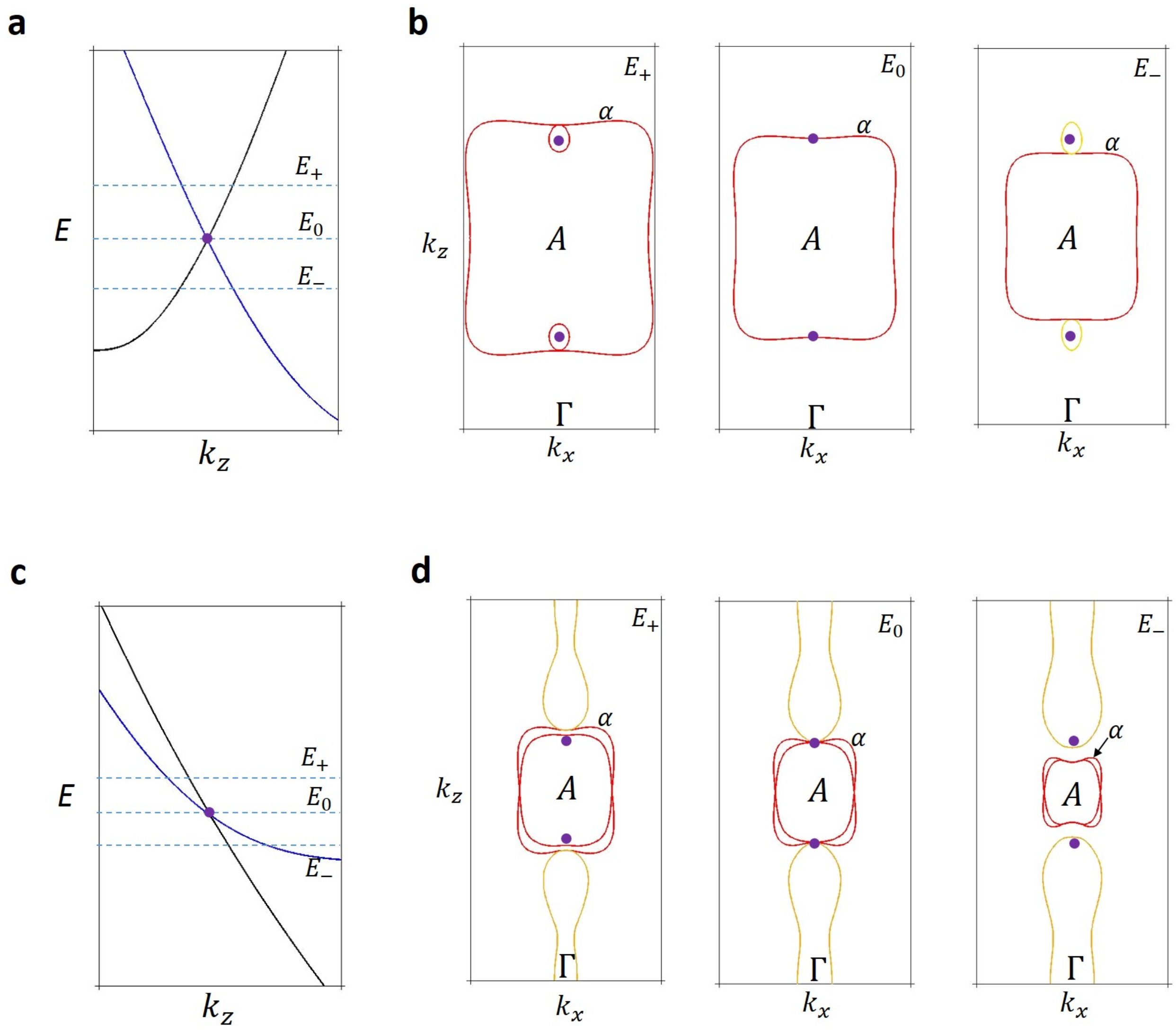

FIG. S1: **Fermiology of the new topological metal.** **a,** Band dispersion around a type-I triply-degenerate node (purple dot). The binding energies of interest are labeled as E_+, E_0 and E_- and marked by blue dashed lines. **b,** Constant energy contour calculations for the (k_x, k_z) surface. At E_0 (middle panel), only one Fermi surface (α-pocket) is observed. At E_+ (left panel) and E_- (right panel), two types of contours are observed. Specifically, two electron pockets (red) are observed at E_+ and one hole pocket (yellow) and one electron pocket is observed at E_-. Due to the doubly-degenerate band along the k_z-axis, the two pockets are always degenerate at a point. **c,** Band dispersion around a type-II triply-degenerate node. **d,** Similar to (b) but for (c). In contrast to the two types of constant energy contours observed in (b), (d) clearly shows three types of constant energy contours, which contain both electron-like and hole-like pockets at E_+, E_0, and E_-. At E_+, two electron pockets (α-pocket) encloses the pair of projected type-II triply-degenerate nodes

FIG. S1: (purple dots) while the hole pocket is degenerate with the outer electron pocket. The calculation at E_0 (middle panel) reveals that three pockets are degenerate at the location of the projected type-II triply-degenerate fermion. At E_- (right panel), the α-pocket is disconnected from the electron pocket and a pair of projected type-II triply-degenerate fermions, while the hole pocket surrounds the projected type-II triply-degenerate fermions.

SI B. Class α triply-degenerate fermions in the WC Family

Except for WC [1] , we have also found other four semimetals, ZrTe [2], δ-TaN [3], NbN [4] and VN [5], which host Class α triply-degenerate fermions. They are all in space group of $P\bar{6}m2$ (187) and have the same crystal structure as WC. The electronic structures of ZrTe, TaN, NbN and VN spin-orbit coupling are plotted in Figs. S2**a,c,e,g**. The energy dispersions in the vicinity of triply-degenerate fermions in ZrTe, TaN, NbN and VN are plotted in Figs. S2**b,d,f,h**. ZrTe, NbN and VN host both type-I and type-II triply-degenerate fermions, while TaN only has type-I triply-degenerate fermions near the Fermi level. The WC family offers a good platform for experimental realization of Class α triply-degenerate fermions for both type-I and type-II.

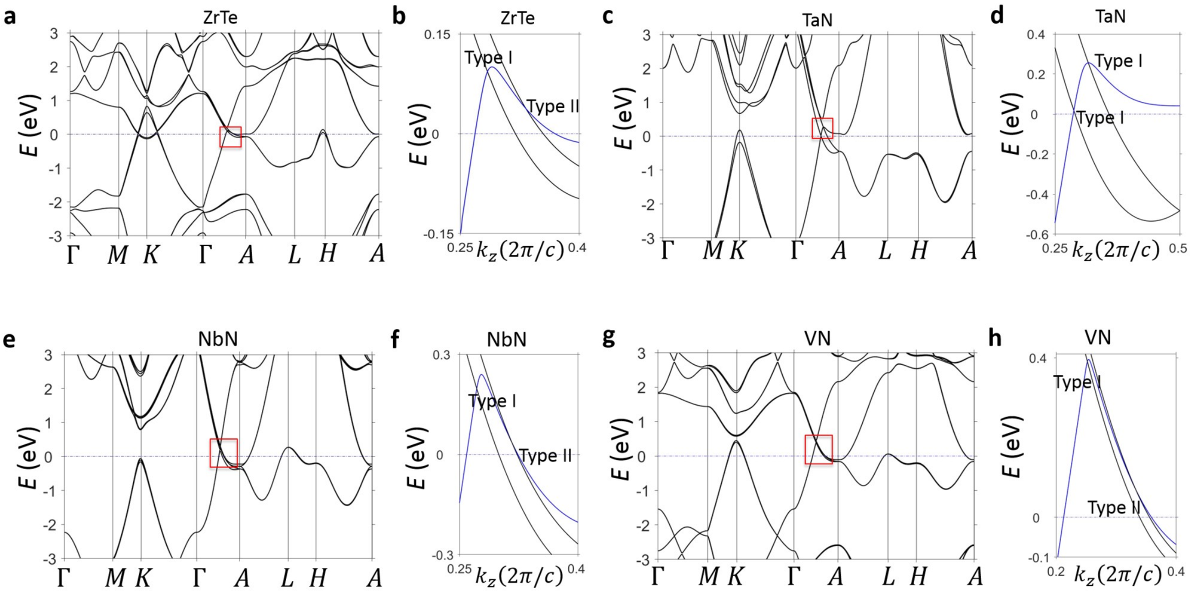

FIG. S2: **Electronic structure of the WC family.** (**a, c, e, g**) Electronic structure of ZrTe, TaN, NbN and VN with SOC. Clear band crossings could be observed aling $\Gamma - A$ in the four compounds. (**b, d, f, h**) The zoom-in view of the band crossings highlighted by the red box in panel (a), (c), (e) and (g). The blue bands are doubly-degenerate.

SI C. Materials hosting Class β triply-degenerate fermions

Here we present two materials that host Class β triply-degenerate fermions. In space group $P3m1$ (156), we found $AgAlS_2$ [10] which contains type-I, Class β triply-degenerate fermions. In space group $P3c1$ (158), we found $RuCl_3$ [6], which has type-II, Class β triply-degenerate fermions.

Material	Space group	Type	Class
WC [1]	187	I and II	I
ZrTe [2]	187	I	I
δ-TaN [3]	187	I and II	I
NbN [4]	187	I and II	I
VN [5]	187	I and II	I
LiScl3 [7]	188	II	I
ϵ-TaN [8]	189	II	I
Li2Sb [9]	190	II	I
AgAlS2 [10]	156	I and II	II
AuCd [11]	157	I	II
RuCl3 [6]	158	II	II
Ge3N4 [12]	159	I and II	II

TABLE S1: **Materials hosting triply-degenerate fermions.**.

1. Type-I, Class β triply-degenerate fermions in AgAlS$_2$

$AgAlS_2$ is in the space group $P3m1$ (156). The crystal structure of $AgAlS_2$ is shown in Fig. S3**a**. The experimental lattice constants, $a = b = 3.5$Å,$c = 6.84$Å, were used in calculations. There are one Ag atom, one Al atom, and two S atoms in the unit cell. The first Brillouin zone of $AgAlS_2$ is plotted in Fig. S3**b**. This lacks the $\mathcal{M}_z$ mirror symmetry. Therefore it is possible to realize Class β triply-degenerate fermions in $AgAlS_2$.

The first-principles calculated band structures of $AgAlS_2$ in the presence of spin-orbit coupling are plotted in Fig. S3**c**. Clear band crossings are observed along $\Gamma - A$, as indicated by the red box. The zoomed-in view of the electronic structures in the red square are plotted in Fig. S3**d**. The two band crossings are both of Class β, type I. To confirm the feature of Class β triply-degenerate fermions, we plotted the energy dispersion in the vicinity of orange dots along k_y direction in Fig. S3**e**. The linear dispersion at the center together with the extra band crossing in the red circle confirm the triply-degenerate fermions in $AgAlS_2$ are Class β.

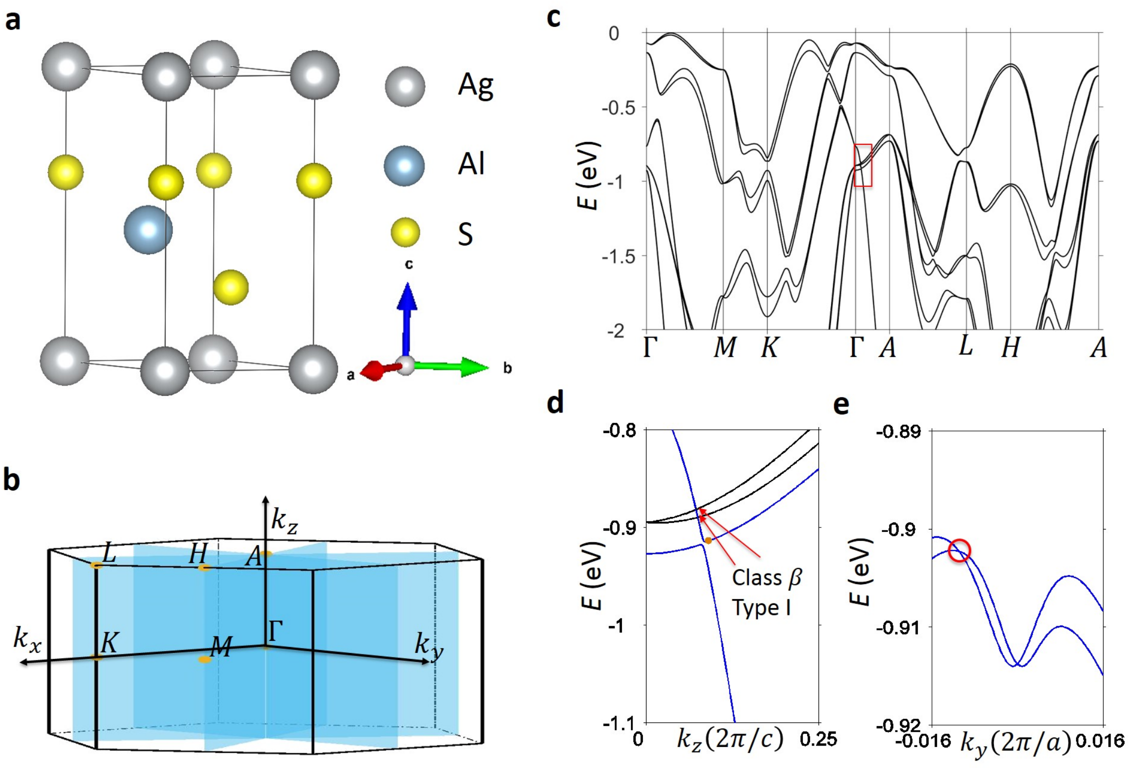

FIG. S3: **Type-I, Class** α **triply-degenerate fermions in** $\mathbf{AgAlS_2}$**.** (**a,**) Crystal structure of $AgAlS_2$ of space group $P3m1$ (156). (**b,**) The bulk Brillouin zone of $AgAlS_2$ with three mirror plane that intersect along the z axis. It lacks of the mirror plane perpendicular to z axis. (**c,**) Electronic structure of $AgAlS_2$ with SOC. (**d,** Zoom-in view of the energy dispersion k_z axis, where the blue bands are doubly-degenerate. Two Type I triply-degenerate fermions are observed. (**e,** The energy dispersion of doubly-degenerate bands along k_y at a generic points (the yellow dots in panel (e)).

2. Type-II, Class β triply-degenerate fermions in $RuCl_3$

The lattice of $RuCl_3$ has the space group $P3m1$ (158), point group C_{3v}. The symmetry of the space group is just enough: a C_3 rotation symmetry along z axis and three mirror planes parallel to z axis. The crystal structure of $RuCl_3$ is shown in Fig. S4**a**. The experimental lattice constants, $a = b = 6.12$ Å,$c = 5.658$ Å, were used in calculations. There are 2 Ru atoms and 6 Cl atoms in the unit cell. The first Brillouin zone of $RuCl_3$ is plotted in Fig. S4**b**.

The first-principles calculated band structures of $RuCl_3$ in the presence of spin-orbit coupling are plotted in Fig. S4**c**. We could observe the band crossings along $\Gamma - A$. The zoom-in view of the electronic structures in the red square are plotted in Fig. S4**d**. The doubly-degenerate blue bands and the two single degenerate black bands have the same sign of velocity. Therefore the triply-degenerate fermions are of type II. We have also plotted the energy dispersion of the blue bands at $k_z = 0.18$ along k_y direction in Fig. S4**e**. From the linear dispersion and the extra band crossings, we confirm that the triply-degenerate fermions in $RuCl_3$ are of Class β.

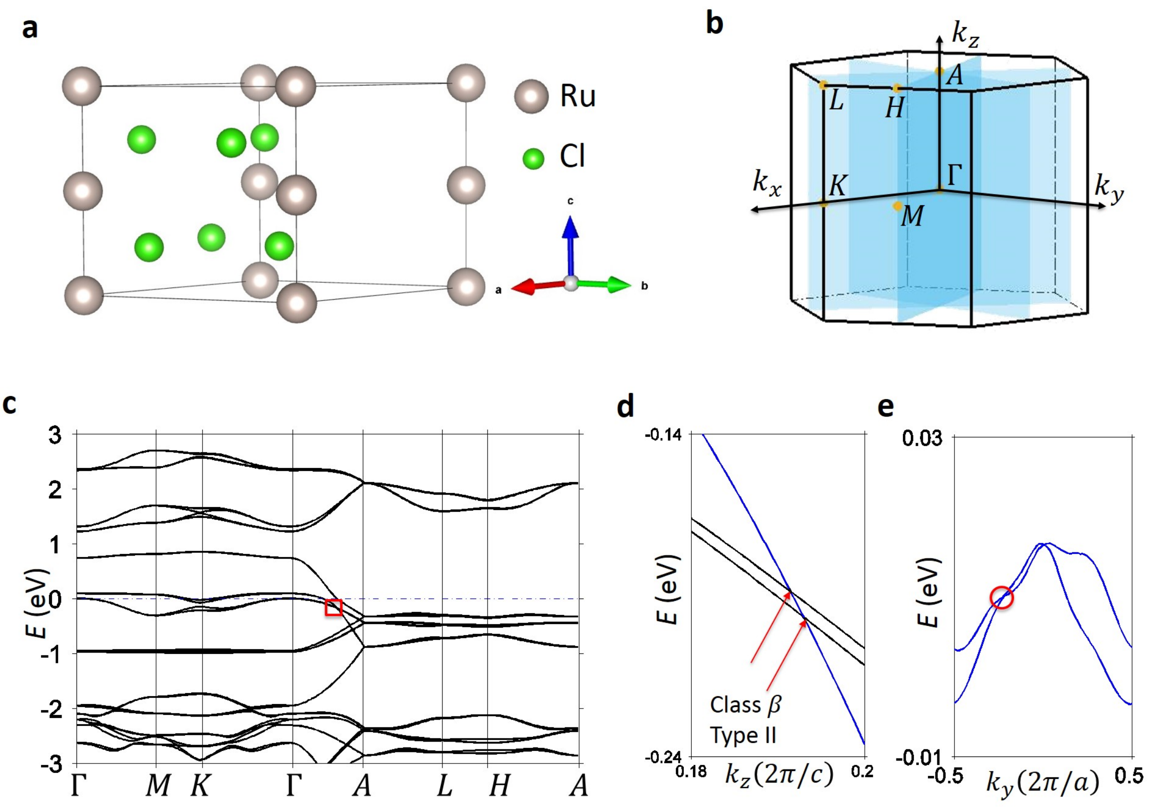

FIG. S4: **Type-II, Class β triply-degenerate fermions in $RuCl_3$.** (**a,**) Crystal structure of $RuCl_3$ in space group $P3m1$ (158). (**b,**) The bulk Brillouin zone of $RuCl_3$. The high symmetry points and planes are highlighted. (**c,**) Electronic structure of $RuCl_3$ with SOC. (**d,** Zoom-in view of the energy dispersion k_z axis. The blue band (doubly-degenerate) and black bands (single degenerate) have the same sign of velocity. (**e,** The linear energy dispersion of doubly-degenerate bands along k_y at a generic points indicates the triply-degenerate fermions in $RuCl_3$ are Class β.

SI D. Effective three-band Hamiltonain in the presence of $\mathcal{M}_z$ symmetry

1. Constructing $k \cdot p$ *Hamiltonain*

The symmetries we will consider are 3-fold clockwise rotation $\mathcal{C}_{3z}$, x-reflection $\mathcal{M}_x$, and $\mathcal{TM}_z$ (combined symmetry of time-reversal and z-reflection). We introduce three basis functions, ϕ_0, ϕ_1, ϕ_2, and they have rotation eigenvalues 1, $e^{-i\frac{2\pi}{3}}$, and $e^{i\frac{2\pi}{3}}$, respectively. When doing x-reflection, $\phi_0 \to \phi_0$, and $\phi_1 \leftrightarrow \phi_2$. As for z-reflection, $\phi_0 \to \phi_0$, $\phi_{1,2} \to \phi_{1,2}$. Here we assume ϕ_0 is $d_{3z^2-r^2}$-like while $\phi_{1,2}$ are $d_{x^2-y^2}/d_{xy}$-like. The three band will constitute the spinless three-fold degenerate Fermi point for ϕ_0 belonging to the singly-degenerate band and ϕ_1 and ϕ_2 to the doubly-degenerate band.

Then we introduce spin-orbit coupling. Owing to we have discussed the symmetry transformations on orbital wave functions, here we illustrate the spin part. For up ($\chi_\uparrow$) and down spin ($\chi_\downarrow$), they transform as $\chi_{\uparrow,\downarrow} \to e^{\pm i\pi/3}\chi_{\uparrow,\downarrow}$ under $\mathcal{C}_{3z}$, and $\chi_{\uparrow,\downarrow} \to i\chi_{\downarrow,\uparrow}$ under $\mathcal{M}_x$ and also under $\mathcal{TM}_z$. Because of the mirror symmetries, one can find that $\psi_1 \equiv \phi_{0\uparrow}$ and $\psi_2 \equiv \phi_{0\downarrow}$ will degenerate as a two-dimensional irreducible representation. Since a linear combination of $\phi_{1\downarrow}$ and $\phi_{2\uparrow}$ is an eigenstate of $\mathcal{C}_{3z}$ symmetry with eigenvalue -1, when they combine as $\psi_{3,4} \equiv \frac{1}{\sqrt{2}}(\phi_{1\downarrow} \pm \phi_{2\uparrow})$, they are also $\mathcal{M}_x$ eigenstates with eigenvalues $\pm i$, which both are one-dimensional irreducible representations. $\psi_{3,4}$ are also eigenstates of $\mathcal{TM}_z$ with eigenvalues $\mp i$.

We will write down the Hamiltonian of the three orbitals–(ψ_1, ψ_2, ψ_3). In this basis, the symmetry operators are

$$\mathcal{C}_{3z} = \begin{pmatrix} e^{i\frac{\pi}{3}} & 0 & 0 \\ 0 & e^{-i\frac{\pi}{3}} & 0 \\ 0 & 0 & -1 \end{pmatrix}, \mathcal{M}_x = -\mathcal{TM}_z = \begin{pmatrix} 0 & i & 0 \\ i & 0 & 0 \\ 0 & 0 & i \end{pmatrix}, \tag{S1}$$

and they require the Hamiltonian satisfying

$$\mathcal{C}_{3z}H(q_+, q_-, q_z)\mathcal{C}_{3z}^{-1} = H(e^{-i\frac{2\pi}{3}}q_+, e^{i\frac{2\pi}{3}}q_-, q_z), \tag{S2}$$

$$\mathcal{M}_x H(q_+, q_-, q_z)\mathcal{M}_x^{-1} = H(-q_-, -q_+, q_z), \tag{S3}$$

$$\mathcal{TM}_z H^*(q_+, q_-, q_z)(\mathcal{TM}_z)^{-1} = H(-q_+, -q_-, q_z), \tag{S4}$$

where $\vec{q}$ is relative to the band crossing point and $q_\pm = q_x \pm iq_y$. By taking $H =$

$\begin{pmatrix} h_{11} & h_{12} & h_{13} \\ h_{12}^* & h_{22} & h_{23} \\ h_{13}^* & h_{23}^* & h_{33} \end{pmatrix}$ and symmetry operators into the above equations, we have, for $\mathcal{C}_{3z}$

$$\begin{pmatrix} h_{11} & h_{12} & h_{13} \\ & h_{22} & h_{23} \\ & & h_{33} \end{pmatrix} (e^{-i\frac{2\pi}{3}} q_+, e^{i\frac{2\pi}{3}} q_-, q_z) = \begin{pmatrix} h_{11} & e^{i\frac{2\pi}{3}} h_{12} & -e^{i\frac{\pi}{3}} h_{13} \\ & h_{22} & -e^{-i\frac{\pi}{3}} h_{23} \\ & & h_{33} \end{pmatrix} (q_+, q_-, q_z), \tag{S5}$$

for $\mathcal{M}_x$

$$\begin{pmatrix} h_{11} & h_{12} & h_{13} \\ & h_{22} & h_{23} \\ & & h_{33} \end{pmatrix} (-q_-, -q_+, q_z) = \begin{pmatrix} h_{22} & h_{12}^* & h_{23} \\ & h_{11} & h_{13} \\ & & h_{33} \end{pmatrix} (q_+, q_-, q_z), \tag{S6}$$

and for $\mathcal{TM}_z$

$$\begin{pmatrix} h_{11} & h_{12} & h_{13} \\ & h_{22} & h_{23} \\ & & h_{33} \end{pmatrix} (-q_+, -q_-, q_z) = \begin{pmatrix} h_{22} & h_{12} & h_{23}^* \\ & h_{11} & h_{13}^* \\ & & h_{33} \end{pmatrix} (q_+, q_-, q_z). \tag{S7}$$

After analysis, the Hamiltonian will look like

$$H(\vec{q}) = t q_z + H_{\mathrm{TB}}(\vec{q}), \tag{S8}$$

where

$$H_{\mathrm{TB}}(\vec{q}) = \begin{pmatrix} \Delta_t q_z & \lambda q_+^2 & \lambda' q_+ \\ \lambda q_-^2 & \Delta_t q_z & -\lambda' q_- \\ \lambda' q_- & -\lambda' q_+ & -\Delta_t q_z \end{pmatrix}. \tag{S9}$$

Here all parameters t, Δt, λ, and λ' are real.

There is a triply-degenerate nodal point located at $\vec{q} = 0$ in the band structure of (S8). Similar to the Weyl (Dirac) semimetals, the degenerate nodal point has important influence on the low-energy electronic properties and can be classified as type-I or type-II [13], depending on the geometry of constant-energy surfaces around it. If $|t| > |\Delta_t|$, the node is type-II; and if, on the other hand, $|t| < |\Delta_t|$, the node is type-I. In the following, we will mainly discuss H_{TB} in Eq. (S9) unless stated otherwise.

To solve the eigenvalues of $H_{\mathrm{TD}}(\vec{q})$ we answer them for $\lambda = 0$ firstly. They are

$$\varepsilon_1^0 = \Delta_t q_z,\ \varepsilon_2^0 = E_n,\ \varepsilon_3^0 = -E_n, \tag{S10}$$

and the corresponding eigenfunctions are

$$\psi_1^0 = \begin{pmatrix} \frac{1}{\sqrt{2}} e^{i\phi} \\ \frac{1}{\sqrt{2}} e^{-i\phi} \\ 0 \end{pmatrix}, \psi_2^0 = \begin{pmatrix} \frac{1}{\sqrt{2}} \cos\frac{\theta_1}{2} e^{i\phi} \\ -\frac{1}{\sqrt{2}} \cos\frac{\theta_1}{2} e^{-i\phi} \\ \sin\frac{\theta_1}{2} \end{pmatrix}, \psi_3^0 = \begin{pmatrix} -\frac{1}{\sqrt{2}} \sin\frac{\theta_1}{2} e^{i\phi} \\ \frac{1}{\sqrt{2}} \sin\frac{\theta_1}{2} e^{-i\phi} \\ \cos\frac{\theta_1}{2} \end{pmatrix}, \tag{S11}$$

where $e^{i\phi} = \frac{q_x+iq_y}{q_\perp}$, $E_0 = \sqrt{2\left(\lambda' q_\perp\right)^2 + \left(\Delta_t q_z\right)^2}$, $\cos\frac{\theta_1}{2} = \sqrt{\frac{1}{2}\left(1+\frac{\Delta_t q_z}{E_0}\right)}$, $\sin\frac{\theta_1}{2} = \mathrm{sgn}(\lambda')\sqrt{\frac{1}{2}\left(1-\frac{\Delta_t q_z}{E_0}\right)}$ with transverse wave number $q_\perp = \sqrt{q_x^2+q_y^2}$. We note that when $\lambda = 0$, the first band is dispersionless in $q_\perp$. Then we introduce λ and we can find the matrix elements of $H_{\mathrm{TB}}(\mathbf{q})$ in the basis $\{\psi_{i=1,2,3}^0\}$ being

$$\left\langle \psi_i^0 \left| H_{\mathrm{TB}}(\mathbf{q}) \right| \psi_j^0 \right\rangle = \begin{pmatrix} \Delta_t q_z + \lambda q_\perp^2 & 0 & 0 \\ 0 & \frac{1}{2}\lambda q_\perp^2 + E_0 - \frac{1}{2}\cos\theta_1 \lambda q_\perp^2 & \frac{1}{2}\sin\theta_1 \lambda q_\perp^2 \\ 0 & \frac{1}{2}\sin\theta_1 \lambda q_\perp^2 & \frac{1}{2}\lambda q_\perp^2 - E_0 + \frac{1}{2}\cos\theta_1 \lambda q_\perp^2 \end{pmatrix}_{ij}, \tag{S12}$$

which indicates the λ term hybridizes ψ_2^0 and ψ_3^0 and keeps ψ_1^0 decoupled. After diagonalizing Eq. (S12), we have eigenvalues

$$\varepsilon_1 = \Delta_t q_z + \lambda q_\perp^2,\ \varepsilon_{2,3} = -\frac{1}{2}\lambda q_\perp^2 \pm \sqrt{2\left(\lambda' q_\perp\right)^2 + \left(\Delta_t q_z - \frac{1}{2}\lambda q_\perp^2\right)^2}, \tag{S13}$$

and their eigenfunctions

$$\psi_1 = \psi_1^0,\ \psi_2 = \cos\frac{\theta_2}{2}\psi_2^0 + \sin\frac{\theta_2}{2}\psi_3^0,\ \psi_3^0 = -\sin\frac{\theta_2}{2}\psi_2^0 + \cos\frac{\theta_2}{2}\psi_3^0, \tag{S14}$$

with $\tan\theta_2 = \frac{\left(\sqrt{2}\lambda' q_\perp\right)\left(\frac{1}{2}\lambda q_\perp^2\right)}{\Delta_t q_z\left(\Delta_t q_z - \frac{1}{2}\lambda q_\perp^2\right) + 2\left(\lambda' q_\perp\right)^2}$. Note that the bands are dispersive in $q_\perp$ for finite λ as is evidenced.

2. Topology

Now we discuss topology in these bands. We calculate the Berry connections for these bands. To make simple, we adopt the differential form notation. The Berry connections are defined as $\mathcal{A}_{ij} = \langle \psi_i | d\psi_j \rangle$ and the Berry curvatures are $\mathcal{F}_{ij} = d\mathcal{A}_{ij}$. We may need elements

as below

$$\begin{aligned}
\left\langle\psi_1^0|d\psi_1^0\right\rangle &= \left\langle\psi_2^0|d\psi_2^0\right\rangle = \left\langle\psi_3^0|d\psi_3^0\right\rangle = 0,\\
\left\langle\psi_3^0|d\psi_2^0\right\rangle &= -\left\langle\psi_2^0|d\psi_3^0\right\rangle = \frac{1}{2}d\theta_1,\\
\left\langle\psi_1^0|d\psi_2^0\right\rangle &= i\cos\frac{\theta_1}{2}d\phi,\\
\left\langle\psi_1^0|d\psi_3^0\right\rangle &= -i\sin\frac{\theta_1}{2}d\phi,
\end{aligned}$$

and hence

$$\langle\psi_1|d\psi_1\rangle = \langle\psi_2|d\psi_2\rangle = \langle\psi_3|d\psi_3\rangle = 0 \tag{S15}$$

$$\langle\psi_3|d\psi_2\rangle = \frac{1}{2}d\theta_1 + \frac{1}{2}d\theta_2, \tag{S16}$$

$$\langle\psi_1|d\psi_2\rangle = i\cos\frac{\theta}{2}d\phi, \tag{S17}$$

$$\langle\psi_1|d\psi_3\rangle = -i\sin\frac{\theta}{2}d\phi. \tag{S18}$$

In above equations, we define $\theta = \theta_1 + \theta_2$ and have used the identities $\left\langle\psi_i^0|\psi_j^0\right\rangle = \delta_{ij}$, $\cos\frac{\theta_{1,2}}{2}d\cos\frac{\theta_{1,2}}{2} + \sin\frac{\theta_{1,2}}{2}d\sin\frac{\theta_{1,2}}{2} = 0$, and $\cos\frac{\theta_{1,2}}{2}d\sin\frac{\theta_{1,2}}{2} - \sin\frac{\theta_{1,2}}{2}d\cos\frac{\theta_{1,2}}{2} = \frac{1}{2}d\theta_{1,2}$. The results send important messages. First, all of three bands are topologically trivial as $\mathcal{F}_{11} = \mathcal{F}_{22} = \mathcal{F}_{33} = 0$. Although the Berry connection is not gauge invariant, the Berry curvature is. Second, to obtain nontrivial topology, hybridizing the bands by breaking symmetry will be needed. Eq. (S16) answers that hybridizing band-2 and band-3 will not produce non-trivial topology. However, Eqs. (S17)-(S18) indicate that hybridizing band-1 and band-2 and/or band-1 and band-3 will be possible since the Chern numbers $\frac{i}{2\pi}\int_S \mathcal{F}_{12} = -\frac{i}{2\pi}\int_S \mathcal{F}_{13} \sim -\frac{1}{4\pi}\int_S d\cos\theta d\phi$ are nonzero, where the integral is over a compact surface enclosing the node. To prove this, we do not show their explicit formulas. Instead, we argue from asymptotic behavior. For a non-trivial homotopic mapping, $(q_x, q_y, q_z) \longmapsto (\phi, \theta)$, there should be a way to make ϕ goes from 0 to 2π and also θ from π to 0, such that $\frac{1}{4\pi}\int_S d\cos\theta d\phi = 1$. We can find that the behavior exists in the wave functions: When $\vec{q}$ circles around the k_z axis, ϕ goes a 2π round too. When q_z goes from $-\infty$ to $+\infty$, θ_2 stays around 0 while θ_1 and hence θ go from π to 0. As a result, a band-crossing node from band-1 and band-2 and a band-crossing node from band-1 and band-3 will be Weyl nodes taking opposite chiral charge.

We give a simple illustration for the above finding. Let's neglect the λ term, which is reasonable when $q_\perp$ is small. In such limit, two Weyl fermions taking opposite chiral charge

will be visible in the Hamiltonian $H(\vec{q})$,

$$H(\vec{q}) = \left(t + \frac{\Delta_t}{3}\right) q_z + \begin{pmatrix} \frac{2}{3}\Delta_t q_z & 0 & \lambda' q_+ \\ 0 & 0 & 0 \\ \lambda' q_- & 0 & -\frac{2}{3}\Delta_t q_z \end{pmatrix} + \begin{pmatrix} 0 & 0 & 0 \\ 0 & \frac{2}{3}\Delta_t q_z & -\lambda' q_- \\ 0 & -\lambda' q_+ & -\frac{2}{3}\Delta_t q_z \end{pmatrix}. \tag{S19}$$

Diffrent from a Dirac fermion composing two Weyl fermions, here, two Weyl fermions as $\mathcal{M}_x$ mirror partners share a common basis. When a Zeeman exchange field along z is added, say the perturbation

$$H_{\text{Zeeman}} = \begin{pmatrix} B_z & 0 & 0 \\ 0 & -B_z & 0 \\ 0 & 0 & 0 \end{pmatrix} \tag{S20}$$

it breaks both $\mathcal{M}_x$ and $\mathcal{TM}_z$ symmetries and will split the triply-degenerate nodal point into three at $q_z = \mp\frac{2B_z}{\Delta t}$ and 0 (relative to the triply-degenerate point). The perturbation will hybridize ψ_1 with ψ_2 and ψ_3 as

$$\langle \psi_i \,| H_{\text{TB}}(\vec{q}) + H_{\text{Zeeman}} |\, \psi_j \rangle = \begin{pmatrix} \varepsilon_1 & \delta h_{12} & \delta h_{13} \\ \delta h_{12} & \varepsilon_2 & 0 \\ \delta h_{13} & 0 & \varepsilon_3 \end{pmatrix}_{ij}, \tag{S21}$$

where $\delta h_{12} = B_z \cos\frac{\theta}{2}$ and $\delta h_{13} = -B_z \sin\frac{\theta}{2}$, which suggests the birth of two Wely nodes taking opposite chiral charge.

SI E. Effective three-band Hamiltonain in the absence of $\mathcal{M}_z$ symmetry

In this section we consider the case without z-reflection symmetry. Since the Hamiltonian in Eq. (S9) does not need to obey Eq. (S4), or Eq. (S7) anymore, the Hamiltonian now becomes

$$H_{\rm NR}(\vec{q}) = \begin{pmatrix} \Delta_t q_z & \lambda q_+^2 + i\lambda_{\rm R} q_- & \lambda' q_+ \\ \lambda q_-^2 - i\lambda_{\rm R} q_+ & \Delta_t q_z & -\lambda' q_- \\ \lambda' q_- & -\lambda' q_+ & -\Delta_t q_z \end{pmatrix}, \tag{S22}$$

in which the Rashba coupling $\lambda_{\rm R}$ appears and λ' could become a complex number. The introduction of the Rashba term will extend the double-band crossing out of the rotation axis k_z. On the mirror plane ($q_x = 0$), the Hamiltonian reads

$$H_{\rm NR}(q_x = 0, q_y, q_z) = \begin{pmatrix} \Delta_t q_z & -\lambda q_y^2 + \lambda_{\rm R} q_y & i\lambda' q_y \\ -\lambda q_y^2 + \lambda_{\rm R} q_y & \Delta_t q_z & i\lambda' q_y \\ -i\lambda' q_y & -i\lambda' q_y & -\Delta_t q_z \end{pmatrix}, \tag{S23}$$

and its eigenvalues are

$$\varepsilon_1(q_x = 0, q_y, q_z) = \Delta_t q_z - \lambda_{\rm R} q_y + \lambda q_y^2, \tag{S24}$$

$$\varepsilon_{2,3}(q_x = 0, q_y, q_z) = \frac{1}{2}\left(\lambda_{\rm R} q_y - \lambda q_y^2\right) \pm \sqrt{2\left(|\lambda'|\, q_y\right)^2 + \left[\Delta_t q_z + \frac{1}{2}\left(\lambda_{\rm R} q_y - \lambda q_y^2\right)\right]^2}. \tag{S25}$$

Owing to opposite mirror parities for band-1 (mirror eigenvalue $m_x = -i$) and band-2,3 (mirror eigenvalue $m_x = i$), two bands will cross on the mirror plane to be line nodes, which positions are determined by $\varepsilon_1 = \varepsilon_2$ (or $\varepsilon_1 = \varepsilon_3$, depending on the sign of q_z). When higher order terms are taken into account, the band-crossing forms a closed loop attaching to the k_z axis on which band-2 and band-3 are degenerate.

SI F. Solutions of Landau Levels

Under an external magnetic field ($\vec{B} = B\hat{z}$), we make the usual Peierls substitution $\vec{q} \to \vec{\Pi} = \vec{q} + \frac{e}{\hbar}\vec{A}$. We neglect the Zeeman splitting since it is typically much smaller than the orbital effect. The Landau level (LL) spectrum and the eigenstates can be obtained by doing the replacement $\Pi_- = \sqrt{2}\ell_B^{-1}a$ and $\Pi_+ = \Pi_-^\dagger$, where the ladder bosonic operators obey $[a, a^\dagger] = 1$ and $\ell_B = \sqrt{\hbar/eB}$. We will introduce the number states, $|n\rangle = \frac{1}{\sqrt{n!}}\left(a^\dagger\right)^n|0\rangle$, leading to $a|0\rangle = 0$, $a|n\rangle = \sqrt{n}|n-1\rangle$, $a^\dagger|n\rangle = \sqrt{n+1}|n+1\rangle$. For this magnetic field, the momentum in $\hat{z}$ remains conserved so q_z will be used to label the eigenenergies and eigenstates. We find that the LLs can be organized into three branches, each labeled by an integer index n.

Guessing the solution

$$\psi_n = \begin{pmatrix} a_n|n+1\rangle \\ b_n|n-1\rangle \\ c_n|n\rangle \end{pmatrix},$$

we come to the eigenvalue problem

$$H_n \begin{pmatrix} a_n \\ b_n \\ c_n \end{pmatrix} = \varepsilon_n \begin{pmatrix} a_n \\ b_n \\ c_n \end{pmatrix}, \tag{S26}$$

where

$$H_n = \begin{pmatrix} \Delta_t q_z & \sqrt{n(n+1)}\gamma & \sqrt{n+1}\omega_c \\ \sqrt{n(n+1)}\gamma & \Delta_t q_z & -\sqrt{n}\omega_c \\ \sqrt{n+1}\omega_c & -\sqrt{n}\omega_c & -\Delta_t q_z \end{pmatrix}, \tag{S27}$$

$\omega_c = \sqrt{2}\lambda'\ell_B^{-1}$ and $\gamma = 2\lambda\ell_B^{-2}$. There are three eigenstates for each n and we use $\alpha = +, -, 0$ to label them. For $\lambda = 0$ the eigenvalues $\varepsilon_{\alpha,n}$ can be expressed analytically. They are

$$\varepsilon_{0,n} = \Delta_t q_z,\ \varepsilon_{+,n} = E_n,\ \varepsilon_{-,n} = -E_n, \tag{S28}$$

and their corresponding eigenfunctions are

$$\psi_{0,n} = \begin{pmatrix} \sqrt{\frac{n}{2n+1}}|n+1\rangle \\ \sqrt{\frac{n+1}{2n+1}}|n-1\rangle \\ 0 \end{pmatrix},\ \psi_{+,n} = \begin{pmatrix} \sqrt{\frac{n+1}{2n+1}}u_n|n+1\rangle \\ -\sqrt{\frac{n}{2n+1}}u_n|n-1\rangle \\ v_n|n\rangle \end{pmatrix},\ \psi_{-,n} = \begin{pmatrix} -\sqrt{\frac{n+1}{2n+1}}v_n|n+1\rangle \\ \sqrt{\frac{n}{2n+1}}v_n|n-1\rangle \\ u_n|n\rangle \end{pmatrix} \tag{S29}$$

for $n \geq 1$ and

$$\psi_{0,0} = \begin{pmatrix} |1\rangle \\ 0 \\ 0 \end{pmatrix}, \psi_{+,0} = \begin{pmatrix} u_0\,|1\rangle \\ 0 \\ v_0\,|0\rangle \end{pmatrix}, \psi_{-,0} = \begin{pmatrix} -v_0\,|1\rangle \\ 0 \\ u_0\,|0\rangle \end{pmatrix} \tag{S30}$$

for $n = 0$, where $E_n = \sqrt{(2n+1)\omega_c^2 + (\Delta_t q_z)^2}$, $u_n = \sqrt{\frac{1}{2}\left(1 + \frac{\Delta_t q_z}{E_n}\right)}$, and $v_n = \mathrm{sgn}(\lambda')\sqrt{\frac{1}{2}\left(1 - \frac{\Delta_t q_z}{E_n}\right)}$. The results show that the 0-th LLs between $\alpha = \pm$ are gapped with $2\omega_c$. We note that the infinite degeneracy in the $\alpha = 0$ band ($\varepsilon_{0,n} = \Delta_t q_z$ regardless of n), which should be limited by the system size, is the artifice of a $q_\perp$-disersionless band in our $k \cdot p$ model.

When $\lambda \neq 0$, all bands in the $k \cdot p$ model become dispersive in $q_\perp$, such that the degeneracy will be lifted. However, the matrix H_n will still show a degenerate eigenstate with energy λ'^2/λ regardless of the value of n when

$$q_z = q_\mathrm{M} \equiv \frac{\omega_c^2}{\gamma\Delta_t} = \frac{\lambda'^2}{\lambda\Delta_t}, \tag{S31}$$

as the determinant of $H_n - E$ is

$$\begin{aligned} \det(H_n - E) &= 0 \\ &= 2n(n+1)\gamma^2\left(\Delta_t q_z - \omega_c^2/\gamma\right) \\ &\quad - (E - \Delta_t q_z)\left[E\left(E - \Delta_t q_z\right) - n(n+1)\gamma^2 - (2n+1)\omega_c^2\right]. \end{aligned}$$

This argument can be verified by diagonalizing H_n for $q_z = q_\mathrm{M}$,

$$E_{0,n} = \lambda'^2/\lambda, \tag{S32}$$

$$E_{\pm,0} = \frac{\lambda'^2}{2\lambda} \pm \sqrt{\frac{\lambda'^4}{4\lambda^2} + n(n+1)\gamma^2 + (2n+1)\omega_c^2}. \tag{S33}$$

This degeneracy of all the middle LLs (i.e. for $E_{n,\alpha=0}$) for $q_z = q_m$ is a result of that when $q_z = q_m$, the middle band of the Hamiltonian (S9) is flat in the transverse $q_\perp$ momentum space. We should not take it too serious as a realistic model might include higher-order terms to split LLs.

Besides, several distinct signatures of the triply degenerate node are discussed. (1) LL spectrum have a triplet structure (with $\alpha = 0, \pm$ for each n). (2) There is LL crossing between the $\alpha = 0$ and $\alpha = \pm$ sets. (3) For $\lambda = 0$, all the LLs with $\alpha = 0$ are degenerate

leading to huge DOS, which potentially will drive the system to broken symmetry states when interaction effects are considered. (4) The collapse of LLs exists for both type-I and type-II triply degenerate nodal points (of model (S9)). In contrast, for Weyl semimetals, the collapse of LLs only appears in for type-II Weyl node [14]. One expects that the magneto-optical response [15] could also exhibit interesting features induced by the extra linear band.

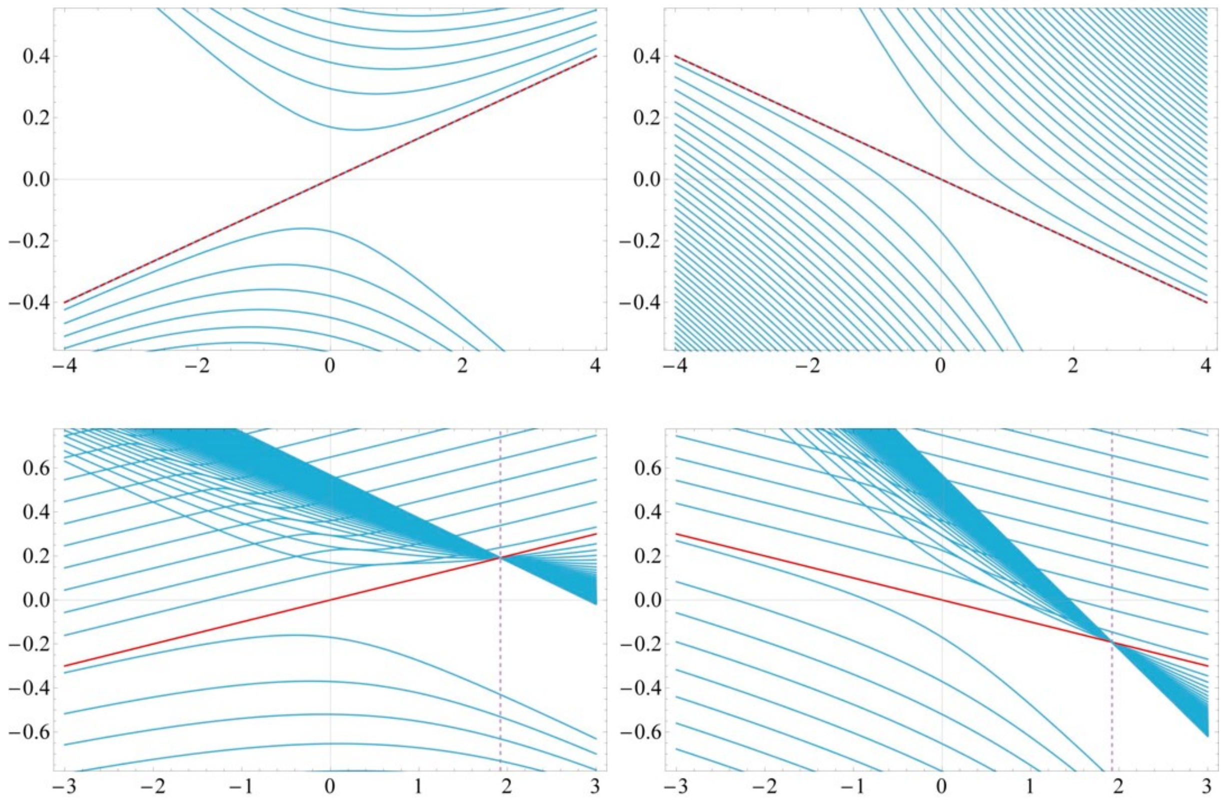

FIG. S5: $\lambda = 0.0$ ($\lambda = 0.05$) for the top (bottom) figures , $\lambda' = 0.12$, $\Delta t = 0.15$, $\ell_B = 1$. Left: type-I node with $t = 0.25$. Right: type-II node with $t = 0.05$. The red lines represent the $alpha = 0$ 0-th LL.

[1] Page, K. *et al.* Reciprocal-space and real-space neutron investigation of nanostructured Mo 2 C and WC. *Solid State Sciences*, **10**, 1499-1510 (2008).

[2] Örlygsson, G. & Harbrecht, B. The Crystal Structure of WC Type ZrTe. Advantages in Chemical Bonding as Contrasted to NiAs Type ZrTe. *Zeitschrift für Naturforschung B*, **54**, 1125-1128 (1999).

[3] Fontbonne, A. & Gilles, J. C. Noveaux nitrures de tantale. Nitrure et oxynitrures mixtes de tantale et de niobium. *Rev. Int. Hautes*, **6**, 181-192 (1969).

[4] Schönberg, N., The tungsten carbide and nickel arsenide structures. Acta Metallurgica, **2**, 427-432 (1954).

[5] Ravi, C., First-principles study of ground-state properties and phase stability of vanadium nitrides. *Calphad*, **33**, 469-477 (2009).

[6] Fletcher, J. M. *et al.* X-Ray, infrared, and magnetic studies of α-and β-ruthenium trichloride. *Journal of the Chemical Society A: Inorganic, Physical, Theoretical*, 1038 (1967).

[7] Lachgar, A. *et al.* Synthesis and properties of two novel line phases that contain linear scandium chains, $LiScI_3$ and $Na_{0.5}ScI_3$. *Inorg. Chem.* **30**, 3321 (1991)

[8] Christensen, A. N. & Lebech, B. A reinvestigation of the structure of ϵ-tantalum nitride. *Acta Crystallographica Section B: Structural Crystallography and Crystal Chemistry*, **34**, 261 (1978).

[9] Müller, W., Notizen: Darstellung und Struktur der Phase Li_2Sb Preparation and Crystal Structure of Li_2Sb. *Zeitschrift fur Naturforschung B*, **32**, 357 (1977).

[10] Range, K. J., Engert, G. & Weiss, A. Darstellung und Kristallstruktur der Hochdruckphase $AgAlS_2$-II/Preparation and Crystal Structure of the High-pressure Phase $AgAlS_2$-II. *Zeitschrift für Naturforschung B*, **29**, 186 (1974).

[11] Alasafi, K. M. & Schubert, K. Kristallstruktur von AuCd. *Journal of the Less Common Metals*, **55**, 1 (1977).

[12] Ruddlesden, S. N. & Popper, P. On the crystal structure of the nitrides of silicon and germanium. *Acta Crystallographica* **11**, 465 (1958).

[13] A. A. Soluyanov, D. Gresch, Z. Wang, Q. Wu, M. Troyer, X. Dai, and B. A. Bernevig, Nature **527**, 495 (2015).

[14] Yu, Z.-M., Yao, Y. & Yang, S. A. Preprint at http://arXiv:1604.04030 (2016).

[15] Malcolm, J. D. & Nicol, E. J., Magneto-optics of massless Kane fermions: Role of the flat band and unusual Berry phase. *Physical Review B* , **92**, 035118 (2015).
